# Multispectral, non-contact diffuse optical tomography of healthy human finger joints

**DOI:** 10.1364/BOE.9.001445

**Published:** 2018-03-02

**Authors:** Daniel Lighter, James Hughes, Iain Styles, Andrew Filer, Hamid Dehghani

**Affiliations:** 1 Sci-Phy-4-Health Centre for Doctoral Training, University of Birmingham, Edgbaston, Birmingham, B15 2TT, UK; 2 School of Computer Science, University of Birmingham, Edgbaston, Birmingham, B15 2TT, UK; 3 Rheumatology, Institute of Inflammation and Ageing, College of Medical and Dental Sciences, University of Birmingham, Edgbaston, Birmingham, B15 2TT, UK

**Keywords:** (170.6510) Spectroscopy, tissue diagnostics, (170.6960) Tomography

## Abstract

Rheumatoid arthritis (RA) is an inflammatory joint disease often affecting the hands, which if untreated causes disability. Diffuse optical tomography (DOT) provides information about the underlying functional properties of biological tissue. To detect pathophysiological changes in inflamed RA joints, a good understanding of the baseline values for healthy subjects is first required. Finger joints from healthy subjects were imaged using a non-contact, multispectral, continuous wave DOT system, recovering physiological parameters of oxygen saturation, total haemoglobin, water concentration and scatter amplitude. Reconstructed values across the cohort demonstrated good consistency between finger joints from the same participant, with greater variation seen between subjects.

## 1. Introduction

Rheumatoid arthritis (RA) is an inflammatory, autoimmune disease of the joints. In humans it is particularly frequently seen in the hands, and if untreated can cause disability. The UK prevalence of RA is 1% [1] and its social impact includes reduced quality of life for patients, increased health-care costs, and working life reduction, with around one third of those diagnosed stopping work on medical grounds within 5 years of initial symptom onset [2]. Despite incomplete understanding of etiology, it is widely accepted that the first three to four months of symptoms provide a window of therapeutic opportunity [3, 4], during which aggressive therapy leads to improved long-term patient outcomes [5]. This creates a clinical need for cheap, non-invasive tools capable of quantifying functional joint changes for early diagnosis and response to therapy. Well established imaging modalities are each subject to specific disadvantages in this regard: radiography suffers from low sensitivity to soft tissue changes occurring in early RA, while both ultrasound and magnetic resonance imaging require highly trained staff leading to high cost and limited availability [6].

Diffuse optical tomography (DOT) is a low cost, non-invasive, non-ionizing and high contrast imaging technique in which near infrared (NIR) light (650 – 930nm) is injected into a tissue at multiple locations on its boundary. Light subsequently exiting the tissue is measured at multiple boundary locations, typically achieved using either optical fibres in contact with the surface of the tissue [7] or a highly sensitive, non-contact imaging device such as a charge-coupled device (CCD) camera [8]. The latter non-contact approach allows greater flexibility in the shape of the imaged tissue and significantly shorter set up time. These multiple measurements are combined to recover a spatial distribution of the underlying optical properties by solving a highly ill-posed and ill-conditioned inverse problem. Three modes of data acquisition are commonly employed in DOT; continuous wave (CW), where only the amplitude of the transmitted light intensity exiting the tissue is measured; frequency-domain (FD), where the mean phase shift for a modulated source is additionally measured; and time domain (TD), which requires measurement of the flight time of individual photons. FD and TD approaches provide additional information about the photon pathlength compared to CW, helping to separate scattering and absorption, however the hardware required for these modes is significantly more expensive than in CW systems.

Relative contributions to optical attenuation from the predominant absorbing chromophores in biological tissue (oxyhaemoglobin (HbO), deoxyhaemoglobin (Hb) and water (H_2_O)), can be spectrally unmixed by collecting data at two or more wavelengths. A spectrally constrained diffuse optical tomography (SCDOT) technique has previously been proposed in which the extinction coefficients, scatter amplitude (S_A_) and scatter power (S_P_) are directly incorporated into the Jacobian matrix, acting as spectral *a priori* information [9, 10]. In addition to recovery of the HbO, Hb and H_2_O, successful recovery of heterogeneities in S_A_ has been demonstrated using CW measurements by optimising the set of chosen wavelengths, [9, 11].

**Fig. 1:**
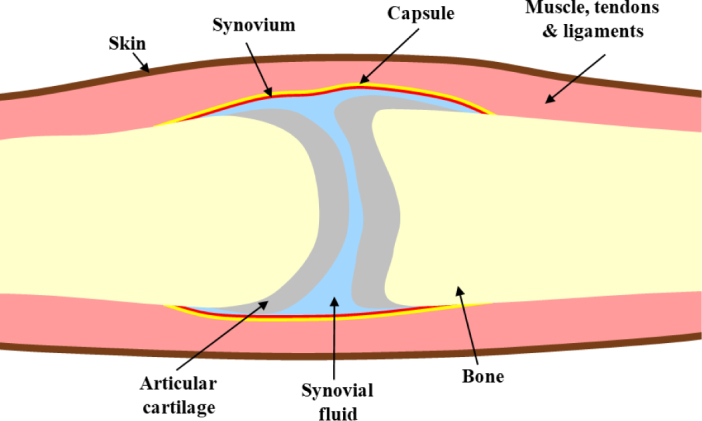
Simplified diagram of structure of the healthy joint.

The physiology of the human finger joint is highly heterogeneous in structure, consisting of 5 main tissue types: bone, cartilage, synovial membrane, synovial fluid and other soft tissues, as shown in Fig. 1. Articular surfaces of the opposing bones either side of the joint are covered in a layer of cartilage. The non-articular surfaces of both bones are connected by a two-layered synovial membrane, which is made up of a thin inner layer called the synovium and a strong outer fibrous capsule layer. This membrane envelops a volume of synovial fluid in the joint cavity and finally soft tissues, including tendon, muscle, ligaments and skin surround this joint structure [12]. In healthy joints, optical contrast is expected in the transverse direction along the finger between the joint cavity and bone either side, with the former exhibiting significantly lower absorption and scattering, which has been reported in previous optical imaging studies [13, 14].

The synovium is a highly specialized tissue, with a primary function of supporting healthy lubrication and nutrition. In afflicted joints of RA patients, a series of signalling pathways stimulate the infiltration of inflammatory cells into the synovium, causing an increased metabolic demand and consequently, a lower localised oxygenation (hypoxia) compared to healthy joints [15]. To meet this increased metabolism, up-regulation of blood vessel formation also occurs (synovial angiogenesis) [16]. These pathological changes lead to altered optical properties of the synovium, with an increase in both absorption and scattering as compared to healthy tissue. Previous studies have used the optical changes in RA as features for classifying diseased joints from healthy. Non-tomographic, single wavelength, CW measurements of transilluminated joints reported achieving this with specificity and sensitivity in the range 80 – 90 % [17], although this relied on comparison with baseline and follow-up examinations of the subjects. A single wavelength, FD DOT system, combined with multivariate machine learning techniques, has been shown to achieve sensitivities and specificities for this application of between 93.8 – 100% [18]. Although effective, FD systems such as this can become prohibitively expensive when developing commercially viable devices.

The focus of this paper is on establishing baseline values and levels of variability in recovered pathophysiological parameters for fingers of healthy controls using a multispectral CW DOT system. The paper is structured as follows: Section 2 presents the developed clinical prototype imaging system; Section 3 outlines the theory behind parameter recovery in finger joints; Section 4 displays typical results from healthy participants; Section 5 contains an analysis of the variation in images of healthy finger joints between subjects; and in Section 6 relevant conclusions and implications for future imaging of RA patients will be discussed.

## 2. Imaging system

**Fig. 2:**
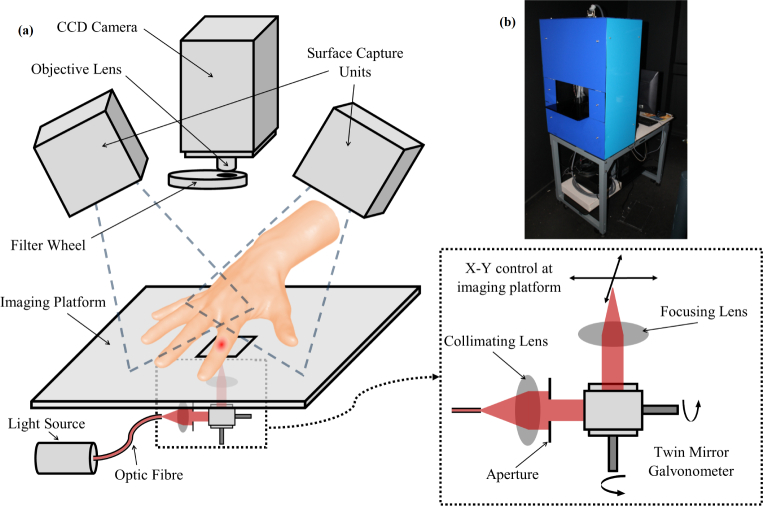
(a) Multispectral DOT imaging system schematic. Insert outlines the non-contact, scanning illumination optical setup. (b) Photograph of the clinical prototype system, fully mobile and suitable for use in the hospital.

The clinical-prototype system used in this presented work is fully non-contact, allowing transmission imaging of joints in the hand, with a system schematic shown in Fig. 2(a). Its development has been based on a previously described first-generation system [19] with a number of modifications which are now described. Multispectral data was collected using an air-cooled back-thinned CCD camera (C4742 - 98, Hamamatsu Photonics), which reduces thermal noise using hermetic vacuum chamber technology. This was coupled to a 25mm/f1.4 focal lens. CCD images were binned into 4×4 square units, resulting in an effective pixel dimension of 0.63 × 0.63 mm at the finger surface. Measurements were spectrally decoupled using a six-position motorised filter wheel (FW102C, Thorlabs, Ely, UK) placed directly in front of the lens, containing a neutral density filter with an optical density of 2.0 and five bandpass interference filters with 10nm FWHM at wavelengths; 650nm, 710nm, 730nm, 830nm and 930nm. This wavelength combination was optimised according to previously outlined criteria, where cross talk between constituent chromophores is minimised and uniqueness of the inverse problem is maximised [9]. CCD images were acquired in a 16 bit format using a high-precision readout mode. An auto-exposure routine was devised to calibrate peak image intensity on the finger to 50,000 counts, thus maximising the signal to noise ratio (SNR) for each wavelength and source position. Furthermore, all raw images were processed by a 5×5 pixel Gaussian filter with a standard deviation of 0.793, based on the estimated FWHM of an equivalent contact-based optical fibre set up, improving the attained SNR.

Illumination of the hand was achieved using a 20W Tungsten Halogen lamp light source (HL2000-FHSA, Ocean Optics, Oxford, UK), coupled to a premium grade, 1000*µ*m diameter optical fibre (QP1000-2-VIS-BX, Ocean Optics, Oxford, UK). By including a 600nm long-pass dielectric filter, the resulting illumination spectrum ranged between 600nm and 2400nm. The inherent spectral response function of the instrument was accounted for through 100 repeat images of a 99% reflectance standard at all 5 wavelengths. A non-contact, scanning illumination system was constructed to allow versatile, rapid and accurate re-positioning of the source position, thus reducing total acquisition times by a factor of approximately 2 compared to the motorized translational stage based system described previously [20]. The optical setup is shown in the insert in Fig. 2(a), where light exiting the optical fibre was collimated using a 25mm/f0.98 plano-convex lens and the diameter of the resulting beam was reduced to 5mm using an adjustable aperture. The direction of this collimated beam was controlled using a dual-axis scanning, silver-coated mirror system (GVS002, Thorlabs, Ely, UK), which was subsequently focused using a 50mm/f1.97 plano convex lens, to create an approximately 1.5mm diameter point source at any arbitrary point within a 40mm × 40mm region on the plane of the imaging platform.

**Fig. 3:**
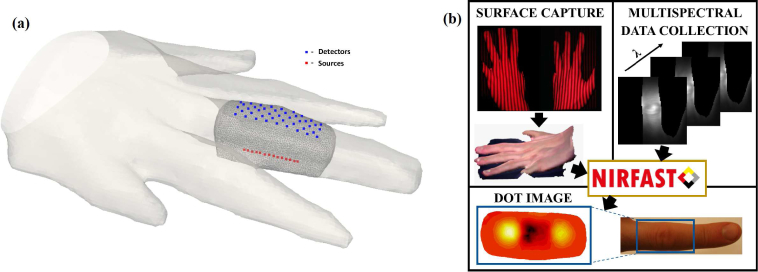
(a) Example of imaged surface profile with corresponding FEM mesh of the finger joint of interest with positions of 14 sources and 43 virtual detectors. (b) Overall work flow for acquiring and processing data.

For tomographic reconstruction, 14 source positions in a straight, central line were used along the underside of the finger with 3mm equal spacing, which provided less variation in intensity and hence good SNR across the finger surface, compared to the possible use of lateral source positions. Additionally, as the underside of the finger is not covered by surface capture data, the assumption that the finger base is at the plane of the imaging platform is more accurate for these chosen central sources. An array of 43 virtual detectors was assigned directly on top of the finger, as shown in Fig. 3(a), which provided a wide field of view. Investigations with higher density arrays (not shown) found limited differences on the reconstructed image quality, therefore a sparser array was chosen to keep reconstruction times short. Increasing the source number will lead to an increase in total acquisition time, whilst a change in the detector number will have no impact, but is limited by the image pixel array. To minimize total imaging time, the source position was switched sequentially after every CCD acquisition at a single filter position, which was then repeated for the remaining wavelengths. A USB-6002 DAQ device (National Instruments, Newbury, UK) was used to both drive the Galvanometer mirror positioning and enable transistor-transistor logic gate control of switching the light source on and off. The surface profile of the hand was acquired in under 20s using two surface imaging units, based on digital fringe projection profilometry [21]. 600nm short pass dielectric filters were added to the projectors in these units, preventing cross talk in the multispectral CCD images. All hardware was controlled via standard computer ports including USB, DVI, HDMI and firewire using LabVIEW (National Instruments, Newbury, UK), on a desktop computer (Stones, UK) with 8GB RAM and an Intel Core i7 CPU 860 at 2.80 GHZ, running 64-bit Windows 7 (Microsoft, Redmond, WA).

A finite element method (FEM) mesh was created from discretizing the volume of interest encapsulated between the finger surface profile and the imaging platform plane lying within a bounding box near the sources, displayed in Fig. 3(a). The resulting 3D tetrahedral mesh typically consists of around 195,000 linear triangular elements which connect around 35,000 nodes, depending on finger dimensions. To register this mesh to the CCD image data, a one time calibration protocol was carried out where a checker-board pattern was simultaneously imaged by both the webcam and the CCD at multiple orientations. Matlab-based automatic multiple camera calibration toolbox [22] was then utilised, which automatically localises checker-board image corners and uses these points to calibrate the respective intrinsic and relative extrinsic parameters of these two cameras. The resulting mapping function was fine tuned by imaging a pattern of white circles on Lego and using a rigid, affine transformation to ensure the circle centroids in the surface profile accurately align with the circle centroids in a corresponding image from the CCD camera.

## 3. Theory

The time independent diffusion approximation, Eq. (1), to the radiative transport equation was used as the forward model for light propagation in biological tissue, when scattering is assumed to dominate over absorption $\left(\mu_{s}^{\prime }\gg \mu_{a}\right)$ and fluence is assumed isotropic after one scattering distance:

$$-\nabla .[D\nabla \phi (r)]+\mu_{a}\phi (r)=S_{0}(r).$$ (1)

where *D*(**r**) = {3[*µ_a_*(**r**) + *µ_s_*(**r**)^′^]}^−1^ is the diffusion coefficient, *S*_0_ (**r**) is the isotropic source, *ϕ* (**r**) is the photon fluence rate and **r** is the position, for a given wavelength *λ*. An index-mismatched type III condition, commonly known as the Robin boundary condition, was used to represent the tissue-air boundary. This forward problem was solved numerically using the FEM mesh generated from surface imaging as a basis over which the problem discretized.

The inverse problem, where an unknown spatial distribution of optical properties is recovered within the finger, was solved using an iterative reconstruction algorithm implemented through open-source FEM package, NIRFAST [23]. This relies on a Newton-like approach to minimize the difference between forward and measured data, whereby differentiating the corresponding objective function results in the following Moore-Penrose generalised update equation:

$$\delta \mu ={\left(J^{T}J+\tau I\right)}^{-1}J^{T}\left[f-F\left(\mu_{a},D\right)\right],$$ (2)

where *f* is the measured boundary data, *F*(*µ_a_*, *D*) is the forward data and *δµ* is the update vector, in our case consisting of *δ*HbO, *δ*Hb, *δ*H_2_O, and *δ*S_A_. At each iteration, optical parameters were interpolated onto a coarse, 3D regular mesh typically made up of ~35,000 linear tetrahedral elements which connect ∼6500 nodes. This coarse mesh was used as a basis in the inverse problem for building J, which is the spectrally constrained Jacobian (also known as the sensitivity matrix):

$$J=\left[\begin{array}{llll} J_{\lambda_{1},c_{1}} & J_{\lambda_{1},c_{2}} & J_{\lambda_{1},c_{3}} & J_{\lambda_{1},S_{A}}\\ J_{\lambda_{2},c_{1}} & J_{\lambda_{2},c_{2}} & J_{\lambda_{2},c_{3}} & J_{\lambda_{2},S_{A}}\\ J_{\lambda_{3},c_{1}} & J_{\lambda_{3},c_{2}} & J_{\lambda_{3},c_{3}} & J_{\lambda_{3},S_{A}}\\ J_{\lambda_{4},c_{1}} & J_{\lambda_{4},c_{2}} & J_{\lambda_{4},c_{3}} & J_{\lambda_{4},S_{A}}\\ J_{\lambda_{5},c_{1}} & J_{\lambda_{5},c_{2}} & J_{\lambda_{5},c_{3}} & J_{\lambda_{5},S_{A}} \end{array}\right],$$ (3)

where $J_{\lambda_{i},c_{j}}=(\delta \theta_{\lambda_{i}}/\delta \mu_{ac_{j}})\cdot \varepsilon_{\lambda_{i},c_{j}}$ and $J_{\lambda_{i},S_{A}}=(\delta \theta_{\lambda_{i}}/\delta D)\cdot (-3D^{2})(\lambda_{i}^{-S_{P}})$, with *δθ* representing log of the boundary intensity. The scatter power, S_P_, was fixed as a homogeneous constant and not updated due to known difficulty in separation with Hb in CW [9]. Tikhonov regularisation was included in Eq. (2), with *τ* initially set to value of 100 ∗ *max*(J^*T*^J) and reduced by a factor of 10^0.25^ every iteration, to speed up convergence. Convergence was achieved when the change in total projection error (the least squared difference between the forward and measured data) was less than 2% as compared to the previous iteration.

A cylindrical, homogeneous, plastic phantom whose optical properties are known *a-priori* [24], was imaged using the system and used to calibrate for the model/data mismatch and any source coupling coefficient variation. A three-step calibration protocol similar to that proposed in previous breast imaging DOT studies [25] was implemented: 1. global values for *µ*_a_ were calculated at each wavelength first using a analytical fit for an infinite medium based on the source-detector separation, followed by a numerical fit using the FEM model (with constant homogeneous $\mu_{s}^{\prime }$ values assumed), 2. using these global values, the data-model offset was calculated as the difference between the measured and modelled data for the homogeneous cylinder, which was averaged for each source position over all detectors, 3. this offset was subtracted from the measured finger data on a source by source basis to produce a calibrated data set. Similar phantom studies to those previously reported for a first-generation system [19] were repeated using heterogeneous, solid plastic phantoms with absorption and scattering anomalies, to ensure the system was able to accurately recover optical properties distributions [26]. These results have not been included for brevity, but are openly available in the supporting data. Initial estimates for the finger were chosen based on literature values, S_A_ and S_P_ set to 2.145 mm^−1^ and 0.77 respectively based on the average of values for bone and muscle [27], and HbO, Hb and H_2_O set to 0.014mM, 0.006mM and 30%, respectively based on previous multispectral images of finger joints [28]. Chromophore extinction coefficients for HbO and Hb are taken from a data compiled by Prahl at the Oregon Medical Laser Center (OMLC) [29] and for H_2_O from data collected by Hale and Querry [30]. Haemoglobin concentrations were then converted to the more physiologically interpretable parameters oxygen saturation (StO_2_) and total haemoglobin (tHb). All data processing was executed in MATLAB (The MathWorks, Natick, Massachusetts, USA), with image reconstruction time typically around 20 minutes, with an accelerated version of the forward model solved in parallel on a GeForce GTX 970 GPU [31], on a desktop computer (Stones, UK) with 16GB RAM and an Intel Core i7 CPU 4790 at 3.60 GHZ, running 64-bit Ubuntu 14.04 LTS.

## 4. Healthy finger imaging

### 4.1. Healthy study design

Ten healthy volunteers (4M / 6F; mean age 27.4 ± 5.6; age range 23 – 39) were enrolled over a one month period, all reporting no previous history of joint disease. Six joints for each participant were imaged, consisting of the 2nd, 3rd and 4th proximal interphalangeal (PIP) joints on each hand, giving 60 finger joints in total. An additional participant (M; age 29) was recruited and imaged consecutively three times, to assess the repeatability of healthy finger data acquired using the system. Ethical approval was obtained from the University of Birmingham (ERN_1_6 – 1490) with all volunteers contacted via word of mouth and required to sign informed consent forms prior to participating.

During an imaging session, the subject rested their hand on the imaging platform, and readjusted its position using a live feed of the CCD to align the PIP joint of the finger of interest with the source positions. Mean total imaging time per joint was 5 minutes, with around 30 seconds for finger repositioning, 20 seconds for surface imaging and around 250 seconds multispectral data acquisition, hence requiring approximately 30 minutes for the full imaging experiment of six joints.

### 4.2. Spectral variation across the joint

**Fig. 4:**
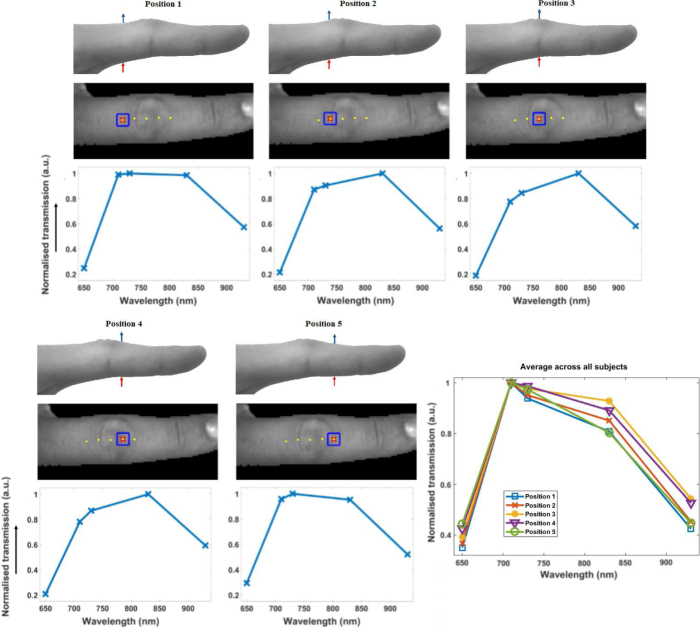
Top row shows the transmission detection (blue) and source (red) setup. Middle row shows finger image for the transmission imaging setup, with all source positions shown as yellow dots, and the current source position underneath the finger (red cross) and detector region directly above the finger (blue square). The corresponding normalised spectral response for that source detector pair is shown on the bottom row. Bottom right graph shows normalised spectral response averaged over all fingers in all ten subjects, for each position.

To illustrate the non-uniformity in spectral response of the raw image data across the joint, image data for five of the source positions were assessed with 9mm spacing across a single joint. The spectral response of the mean transmitted intensity within a region of interest of 8 × 8 pixels (2.5 × 2.5mm on the finger surface) in the images, which lies directly above the corresponding source position, is shown in Fig. 4.

The example spectral response curves shown for one finger in Fig. 4 demonstrate evidence of a distinction between position 3, predominantly expected to sample the joint cavity, compared to positions 1 and 5, where the transmitted volume will mainly contain bone and soft tissues. Compared to positions 1 and 5, the spectra at position 3 shows comparatively greater relative transmission at higher wavelengths 830nm and 930nm compared to the lower wavelengths 650nm, 710nm and 730nm. This pattern is again evident when comparing similar regions averaged over all fingers in all subjects, with greater relative transmission at the higher wavelengths for positions 3 and 4, as shown in Fig. 4. This illustrates the additional information obtained from collection of data at multiple wavelengths, which can be used to distinguish the pathophysiological differences between the joint cavity and the surrounding bones.

### 4.3. Typical in vivo DOT images

**Fig. 5:**
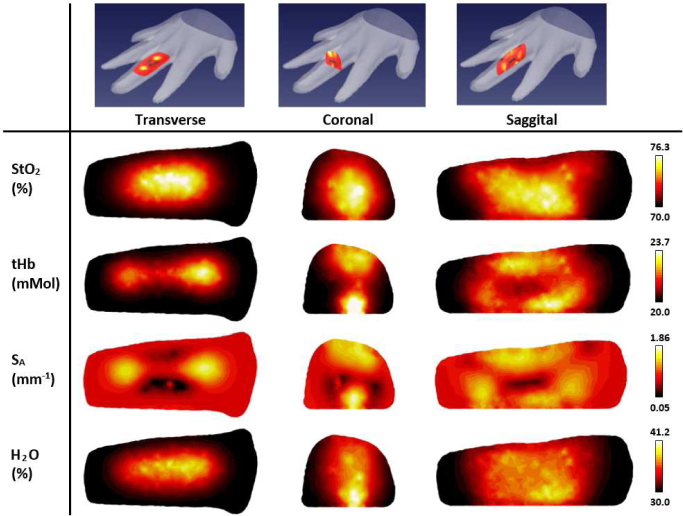
Example reconstructed images for a single healthy participant. The top row shows the captured surface profile of the hand overlayed with three perpendicular slices considered for visualisation. Corresponding image slices are displayed below of recovered parameters oxygen saturation (StO_2_), total haemoglobin (tHb), S_A_, and H_2_O.

A typical set of image slices from a healthy PIP joint for the coronal, transverse and sagittal planes are displayed in Fig. 5 with the corresponding orientations of these slices also shown by overlaying example slices of S_A_ on the hand surface profile. We show reconstructed image maps of the clinically relevant parameters StO_2_, tHb, S_A_ and H_2_O.

The recovered contrast is most qualitatively apparent for S_A_, with very low values of down to 0.05mm^−1^, recovered in the centre of the transverse and sagittal images, consistent with findings in previous literature [13, 28]. This region is expected to correspond to the joint cavity, where a key contributing factor to this contrast is attributed to the presence of synovial fluid, which is highly transparent and has low scattering. Regions either side of this with high S_A_ up to 1.865mm^−1^ are expected to be due to the bones on either side of the joint cavity, as bone is known to be highly scattering [27]. Lower concentrations of tHb are also observed in the central regions of the transverse and sagittal images, although the contrast between the joint cavity and bone regions is much less significant, with values for the example in Fig. 5. ranging between 20.0 and 23.7 mMol. The linear relationship between chromophore concentration and absorption coefficient means this region of lower tHb would imply lower absorption coefficients in the centre relative to the surrounding bones, also consistent with findings in previous literature [13, 28]. The epiphysial-metaphyseal (end regions) of the bone and the synovium are highly vascularised, whilst the synovial fluid has an absence of direct blood supply and the articular cartilage should be avascular to retain its mechanical performance [32], which may explain this observed contrast along the joint. Neither H_2_O nor StO_2_ images reveal any discernible information regarding the joint structure, with the distribution of these parameters much more homogeneous compared to S_A_ and tHb.

## 5. Healthy subject variation

For visualisation of all imaged joints in Fig.s 6–9, slices were taken along the central transverse plane of the joint, as illustrated in Fig. 5. A delineation of the joint cavity from the surrounding bone is consistently visible for all fingers, with a central region of lower S_A_ and tHb compared to either side. This contrast is consistently most pronounced in S_A_ images, with an S_A_ of around 2mm^−1^ in the region expected to reflect bone, whilst some very low scattering is measured in the joint cavity, approaching 0.01mm^−1^. All reconstructed images of StO_2_ and H_2_O shown in Fig.s 6 and 9 do not demonstrate the same evidence of joint structure as in tHb and S_A_. Some fingers are showing a recovered oxygenation lower than the initial estimate, as low as 62%, whilst other fingers give a recovered oxygen saturation of up to 82%. This variation may be the result of a natural variability in the oxygenation between participants or it may be a result of amplification of measurement error due to StO_2_ being computed from the division of HbO by tHb. Further work should investigate whether chromophore concentrations HbO and Hb are better biomarkers for RA classification than StO_2_ or tHb. Qualitatively, PIP joint images for participants A-J in Fig.s 6–9. appear more similar between fingers from the same participant compared to images from different participants. For example, contrast in all reconstructed parameters appears consistently lower for participants F and I compared to the other participants.

**Fig. 6:**
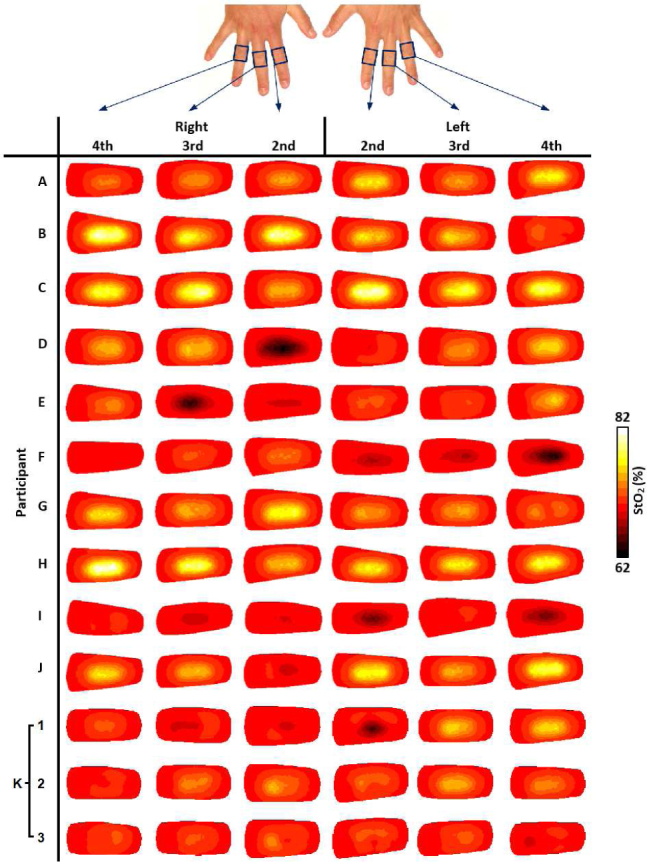
Transverse slices half way through the finger of StO_2_ (%), for all imaged joints.

**Fig. 7:**
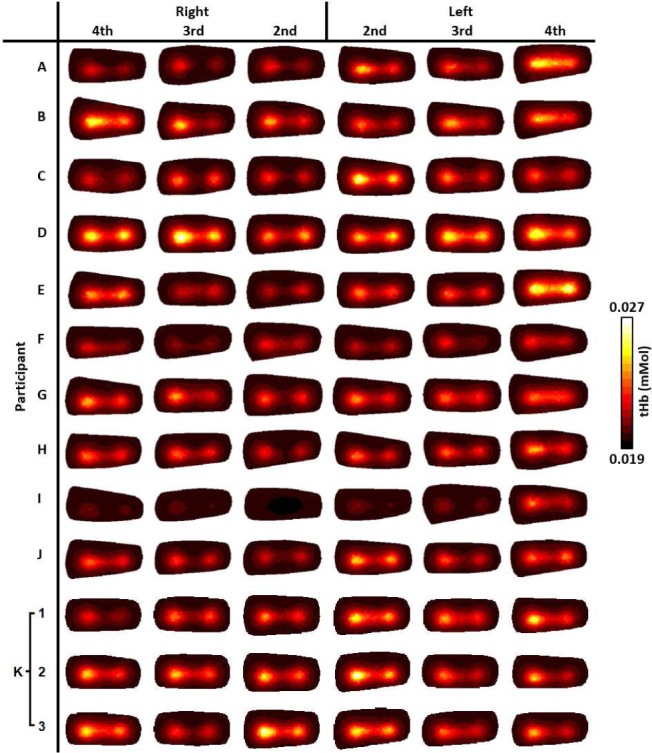
Transverse slices half way through the finger of tHb (mMol), for all imaged joints.

**Fig. 8:**
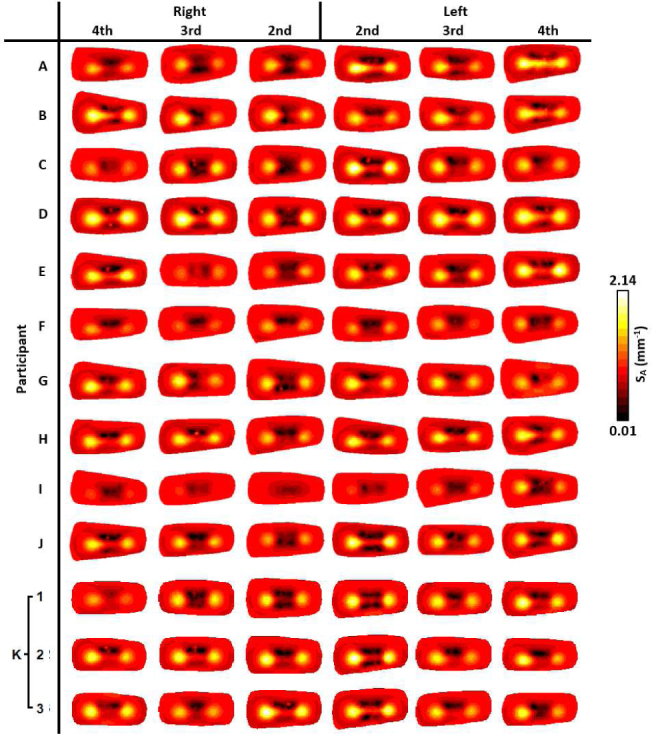
Transverse slices half way through the finger of S_A_ (mm^−1^), for all imaged joints.

**Fig. 9:**
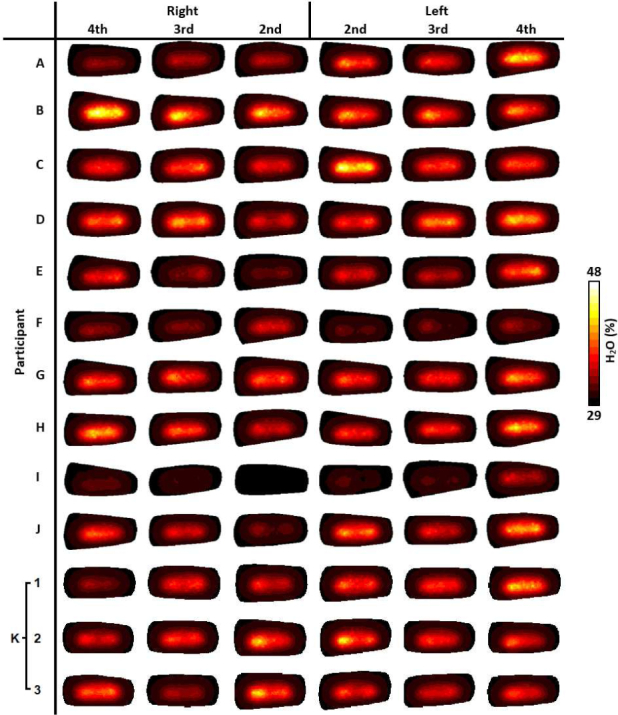
Transverse slices half way through the finger of H_2_O (%), for all imaged joints.

The three repeat measurements of participant K are also shown in Fig.s 6–9. These appear qualitatively more similar than when compared to other participant finger sets, however some variation between repeats is evident, particularly for StO_2_ images of 2nd and 4th PIP joints of the left hand, ranging between around 65% to 75%, again indicating that this parameter is particularly sensitive to measurement errors. Data acquisition on tissue-mimicking phantoms (not shown) using the system was found to be highly repeatable. Potential sources of variability when repeating imaging of human joints can include variation in the initial hand positioning from a live feed of the CCD camera which could impact source coupling, or any movement during data acquisition, which will be subject of future studies including methods for minimising such errors.

**Fig. 10:**
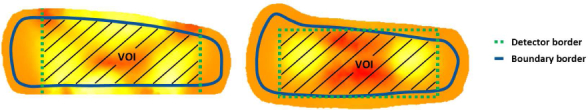
Volume of interest (VOI) only included nodes lying directly below the detector grid and ignoring 50% of nodes closest to the boundary. Examples of the cut off border lines shown for both a sagittal slice (left) and a transverse slice (right) on an S_A_ image.

**Fig. 11:**
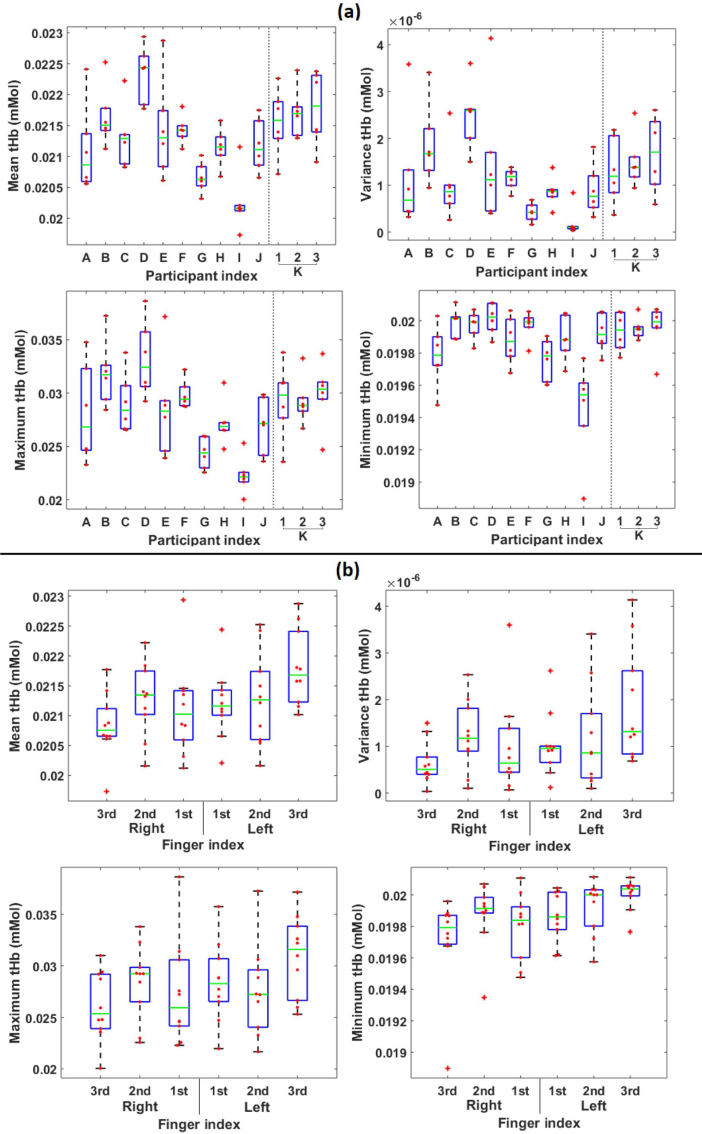
Box plots of tHb with (a) each group containing values for all six fingers from the same participant, for participants A - J and three repeats of participant K and (b) each group containing values from participants A - J for the same finger index. All four image features are plotted, displaying the median (green line), interquartile range (blue box), range (black) and outliers (red cross), overlayed with a beeswarm plot of all data points (red dot).

For quantitative assessment of the metabolic images, the features: mean, variance, maximum and minimum; were calculated for nodes within a volume of interest (VOI), as shown in Fig. 10. The VOI was defined to only include nodes lying directly below the detector grid, as low sensitivity outside this volume means optical properties were not updated. Additionally, the VOI ignored 50% of nodes closest to the boundary, as it is known that hypersensitivity close to the sources and detectors can amplify errors in this region [33]. To illustrate the variance in these image features for healthy subject data, box plots overlayed with beeswarm plots of tHb are displayed in Fig. 11.

The variance in image feature values across fingers within individual participants A-J, shown in Fig. 11(a), is generally smaller than the variance across participants for a given finger index, shown in Fig. 11(b), indicated by the narrower interquartile ranges and total ranges seen. Additionally, the median values of each finger index for the ten participants in Fig. 11(b) are more similar than the median values of each participant for all six fingers in Fig. 11(a). Boxplots of the three repeats for participant K show similar median values and variance to one another, for all image features. Due to space considerations, boxplots for S_A_, H_2_O and StO_2_ have not been shown, however these also display similar trends in distributions to those discussed for tHb.

Statistical analysis of levels of variability was carried out using the Kruskall-Wallis (K-W) test, with a null hypothesis that the median of all groups are the same. Groups chosen for comparison were; “different subjects” with each group containing all fingers for each of the individual participants A-J, “different fingers” with each group containing all participants A-J for a particular finger index, and finally “different repeats” with each group containing all fingers for every repeat data set for participant K. In all comparisons, differences between groups were taken as significant if p was less than 0.05.

The resulting p-values for all K-W tests are displayed in Table 1. The null hypothesis for variability between different subjects is rejected for all features except minimum S_A_, with the majority of p-values much less than 0.05, meaning a statistical difference is seen between different participants in recovered pathophysiological parameters. In contrast, the null hypothesis is accepted for all features for variability between fingers, with the exception of minimum tHb. These two results will be due to a combination of: low variation between joints of the same participant, and high variation between joints of different participants. The null hypothesis is also accepted for all features when comparing the three repeat measurements of participant K, demonstrating that the system is capable of recovering similar pathophysiological values from a subject over multiple imaging sessions. The current methodology appears to be able to discriminate between healthy participants, with variation between subjects more significant than variation between fingers or between repeat measurements, hence demonstrating that the system is capable of consistently recovering similar values within joints of the same participant.

**Table 1:** p-values for the Kruskall-Wallis test assessing variance between different subjects, different fingers and different repeats, displayed for all four image features; mean, variance, maximum and minimum, and all four metabolic parameters. Significant values are marked ^*^.

	Different subjects				Different fingers				Different repeats			
	Mean	Var	Max	Min	Mean	Var	Max	Min	Mean	Var	Max	Min
StO_2_	0.000014^*^	0.00049^*^	0.000011^*^	0.000030^*^	0.87	0.73	0.80	0.79	0.23	0.49	0.57	0.46
tHb	0.000073^*^	0.000094^*^	0.000080^*^	0.0011^*^	0.062	0.11	0.17	0.01^*^	0.71	0.64	0.65	0.65
S*_A_*	0.000028^*^	0.00017^*^	0.00014^*^	0.063	0.17	0.43	0.32	0.94	0.53	0.65	0.52	0.33
H_2_O	0.000063^*^	0.000061^*^	0.000023^*^	0.0000057^*^	0.20	0.16	0.31	0.92	0.88	0.99	0.93	0.98

## 6. Discussion

Achieving high sensitivities and specificities when classifying between diseased and healthy joints will rely on a combination of a low variation within each class and a large discrepancy between the two classes. We concern ourselves here with assessing baseline variability in healthy participants, to help understand the consistency of recovered parameters and identify those that may prove potentially useful for classification in future studies including patients with arthritis.

In the presented data, images recovered for scattering amplitude and total haemoglobin from human hand joints of healthy participants demonstrate a consistent distribution, with central regions of lower S_A_ and tHb. This contrast agrees well with the expected optical contrast previously reported in the literature, with regions of lower values expected to reflect the joint cavity compared to relatively greater values either side, anticipated to correspond to bone. We hypothesize that in RA patients, the quantitative contrast between the joint cavity and the bone will reduce, as both scattering increases, due to a greater number of inflammatory cells, and local blood volume fraction increases, resulting from synovial angiogenesis, in the inflamed joint cavity. In these images, parameters StO_2_ and H_2_O are not able to distinguish the joint structure with the same clarity, however quantitatively these parameters are both consistent at around 72% and 33% and may still prove useful for classification if a large difference is seen in images of arthritis patients, such as significant hypoxic conditions [15] or bone marrow oedema [34]. Evaluation of the significance of the variability observed in this healthy subject study for diagnosing RA, either between different subjects, different fingers or repeats, requires a knowledge of the typical ranges of values for patients with RA, which will be determined in future studies.

In healthy participants, variation between different finger indices is statistically less variable than the variation between participants. This indicates that the outlined system and data recovery algorithm is more consistently capable of recovering useful parameters from different joints of the same participant than across joints from different participants. This is perhaps not surprising if metabolic parameters are expected to be more consistent within the hands of one person experiencing the same physiological conditions. It also must be acknowledged that absolute values recovered in optical imaging are often influenced by other factors, for example the structure of the finger joint may introduce quantitative discrepancies, an effect which would be less significant between fingers from the same person with similar sizes and underlying anatomy. High levels of inter-subject variation are widely acknowledged in recovery of absolute parameters using optical imaging. For example, this was reported for tHb when recovering absolute values with DOT studies of the breast [35, 36]. As the severity of inflammation is typically not uniform across all joints in the hands of an RA patient, with certain joints more severely afflicted than others [37] (although a degree of symmetry is commonly seen), the level of variability across fingers of the same subject should increase in patients with joint inflammation compared to healthy subjects.

To conclude, results from a healthy cohort study have been presented, demonstrating the capability of a multispectral CW DOT system to recover pathophysiological parameters which consistently delineate the joint cavity from surrounding bone for 66 pip joints, including 3 repetitions of 6 joints, all images of which are qualitatively in good agreement with optical measurements reported previously in the literature. Furthermore, statistical analysis indicates that the system is more consistent between different finger joints within the same subject, than for the same finger joints from different subjects. Future work will include a larger cohort study including both healthy subjects and arthritis patients.
